# Detection and Characterization of Feline Calicivirus Associated with Paw and Mouth Disease

**DOI:** 10.3390/ani13010065

**Published:** 2022-12-23

**Authors:** Andrea Palombieri, Vittorio Sarchese, Maria Veronica Giordano, Paola Fruci, Paolo Emidio Crisi, Giovanni Aste, Laura Bongiovanni, Valentina Rinaldi, Alessio Sposato, Michele Camero, Gianvito Lanave, Vito Martella, Fulvio Marsilio, Barbara Di Martino, Federica Di Profio

**Affiliations:** 1 Department of Veterinary Medicine, Università degli Studi di Teramo, 64100 Teramo, Italy; 2 Department of Biomolecular Health Sciences, Faculty of Veterinary Medicine, Utrecht University, 3584 Utrecht, The Netherlands; 3 Department of Veterinary Medicine, Università Aldo Moro di Bari, 70121 Bari, Italy

**Keywords:** FCV, paw and mouth disease, immunohistochemistry, WGS, phenotypic and antigenic characterization

## Abstract

**Simple Summary:**

Feline calicivirus (FCV) is a common viral pathogen affecting domestic cats, which is responsible for diverse clinical presentations, commonly including upper respiratory tract signs, oral ulcerations, and a fever. In addition, FCV infection can be associated with severe pneumonia, lameness, and virulent systemic disease. In this study, we clinically and pathologically describe a rare case of FCV-associated paw and mouth disease in a febrile household cat. The FCV strains detected in the animal and in an overtly healthy cohabiting cat were analyzed to assess the phenotype and antigenic properties.

**Abstract:**

Feline calicivirus (FCV) infection in cats can led to several diverse clinical presentations, ranging from mild upper respiratory signs to virulent systemic disease. Herein, we report a paw and mouth disease case in a 7-year-old household cat due to an FCV infection. An asymptomatic cat living in the same household was also infected with FCV. Clinical and pathological investigations were combined with the molecular and phenotypical characterization of the FCV strains. The RNA of the FCV was detected using qualitative and quantitative reverse transcription (RT)-PCR assays, and FCV antigen was confirmed by immunohistochemistry. After the whole genome analysis, the strains detected in the two cats appeared to be genetically diverse from FCVs previously detected in association with paw and mouth disease and with virulent systemic disease. Interestingly, the isolates obtained in this study were resistant to low pH conditions and slightly susceptible to bile salts, but they were susceptible to a trypsin treatment, revealing a phenotype pattern that is different from that which has been observed for respiratory FCVs.

## 1. Introduction

Feline calicivirus (FCV), a member of the genus *Vesivirus* in the family *Caliciviridae*, is a small (~35–40 nm), icosahedral, non-enveloped virus that shares a characteristic morphology with approximately 32 cup-like depressions in an icosahedral symmetry. The viral genome is a positive-sense, single-stranded RNA molecule of about 7.7 kilobases (kb) in length, and it is predicted to encode three open reading frames (ORFs) [1]. ORF1 encodes a 200 kDa polyprotein that is processed by a viral proteinase to produce several nonstructural proteins [2,3], ORF2 encodes a 73-kDa capsid precursor protein (pre-VP1) that is post-translationally processed to release the 60 kDa mature capsid protein VP1 [4], and ORF3 encodes a small basic protein (VP2) with a predicted molecular weight of 12 kDa, which has been associated with the stability of the capsid [5,6].

FCV is a highly contagious pathogen with a widespread distribution in the general cat population. The virus was originally isolated in New Zealand in 1957 [7] from the intestinal content of cats showing upper respiratory symptoms, oral ulcerations, and a fever. Since then, FCV infection has been associated with a variety of clinical signs, including acute febrile lameness syndrome, abortion, severe pneumonia, and acute enteritis [8,9,10,11]. Furthermore, in the last two decades, there has been an increasing number of reports of virulent FCV strains, causing outbreaks of severe virulent systemic disease (VSD) with a high mortality [12,13,14,15,16,17,18,19,20,21,22,23]. The clinicopathological features of VSD-FCV differ substantially from those of “classical” FCV disease. The characteristic signs include a high persistent fever, marked subcutaneous oedema, mainly located on the limbs and face, ulcerative dermatitis, icterus, pulmonary oedema, and coagulation abnormalities [12,14,15]. Before the description of VSD-FCV [12], a paw and mouth disease (PMD) characterized by oedema and ulcerative lesions, mainly located on the limbs, head, and mouth had been described in Australia [24,25], and since then, it has been sporadically reported in the USA and Europe [26,27]. Although, the initial clinical presentation of the disease is similar to that reported for VSD, inner organ involvement and high mortality have not been observed in PMD. Additionally, in contrast to VSD-FCV, PMD has been reported in either single cases or in very small outbreaks, without an epizootic course [28].

Herein, we report the clinical and pathological characterization of a case of PMD associated with FCV infection in a household cat. The genome of the FCV strain was sequenced, and its phenotype and antigenic properties were assessed.

## 2. Materials and Methods

### 2.1. Case Description

**Figure 1:**
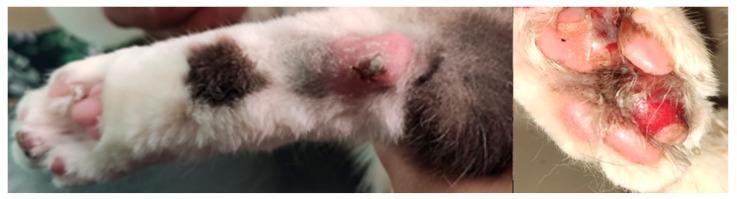
Severe ulcerative and necrotizing pododermatitis with serocellular crusts.

A 7-year-old male neutered domestic shorthair cat (#9284-5) was presented to the Veterinary Teaching Hospital at the Faculty of Veterinary Medicine, University of Teramo (Teramo, Italy) in July 2021 after 2 days of inappetence, lethargy, and lameness. The cat lived exclusively indoors along with a 9-year-old female cat (#9341-1) that did not present any clinical signs. Both of the cats received the last vaccination for feline panleukopenia, rhinotracheitis, and calicivirus in August 2018. Upon physical examination, the cat was febrile (40.7 °C) and showed painful swelling of all of its footpads, which over the next 2 days, progressed to erosion and ulceration (Figure 1), associated with the appearance of limb and tongue ulcerations.

At the time of admission, a moderate lymphocytopenia (0.59 K/μL; reference interval (RI) 0.92–6.88 K/μL) and eosinopenia (0.00 K/μL; RI 0.17–1.57 K/μL) were noted on the complete blood count, while the serum chemistry revealed increased activities of creatine kinase (624 U/L; RI 91–326 U/L), alanine aminotransferase (47 U/L; RI 22–45 U/L), and aspartate aminotransferase (44 U/L; RI 14–41 U/L). The cat tested negative for FIV and FeLV on a point-of-care (POC) immunochromatographic test (SNAP FIV/FeLV Combo Test, IDEXX) (IDEXX Laboratories, Inc., Westbrook, ME, USA). Plain radiography of the thorax and abdominal ultrasound were within normal limits. A biopsy specimen was collected at the edge of a footpad ulcerative lesion by using an 8 mm biopsy punch, which was processed for paraffin sectioning and stained with haematoxylin and eosin. The histological examination revealed a severe, diffuse, ulcerative pododermatitis, which was characterized by full-thickness necrosis of the epidermis and dermal granulocytic and lympho-histiocytic inflammation. No fungal elements or bacteria were evident. Additional samples of the affected tissues were collected using cytobrushes, along with blood and serum samples and oropharyngeal and rectal swabs for virological investigation. The sample collection also included an oropharyngeal specimen and a serum sample obtained from the asymptomatic cat living with the diseased animal. Informed consent was obtained from owner of the cats. The presence of common feline viral pathogens including FCV, feline herpesvirus type 1 (FHV-1), feline panleukopenia parvovirus (FPV), and feline coronavirus (FCoV) was investigated either by quantitative (real-time) PCR (qPCR) or qualitative PCR after reverse transcription (RT) [29,30,31,32]. The serum and blood samples were also tested for FeLV and FIV pro-viral DNAs [33,34].

The cat, #9284-5, was hospitalized, and enteral nutrition was guaranteed via a rhino-esophageal feeding tube; the patient was treated with prednisolone 1 mg/kg q24h, subcutaneously, buprenorphine 20 μg/kg q8h EV, and doxycycline oral paste, which were given through feeding tube at the dosage of 10 mg/kg once a day. In addition, a chlorhexidine-based odontostomatological gel was administered once a day. The cat became normothermic after 3 days and restarted to eat voluntarily. After one week, the patient was discharged from the hospital, and the prednisolone treatment was gradually tapered off. Two weeks later, the cat was presented for a control visit, and at the physical examination, the complete healing of the ulcerative lesions was observed. Additionally, the oropharyngeal swabs of both of the cats were negative for FCV on the qualitative and quantitative RT-PCR.

### 2.2. Real Time RT-PCR (RT-qPCR) for FCV 

The FCV RNA quantification was performed using the SuperScript III platinum OneStep Quantitative RT-PCR system (Invitrogen Ltd., Milan, Italy) in a 25 μL volume comprising 5 μL of extracted RNA and 20 μL of master mix. The primers (FCV For: 5′-GTTGGATGAACTACCCGCCAATC-3′ and FCV Rev: 5′- CATATGCGGCTCTGATGGCTTGAAACTG-3′) and a TaqMan probe (FCV Probe: 5′-TCGGTGTTTGATTTGGCCTG-3′) [35,36] were used at concentrations of 200 and 100 nM, respectively. A standard curve was generated using tenfold serial dilutions (from 10^9^ to 10^0^ copies per reaction) of FCV plasmid DNA, which were constructed by cloning 83 bp ORF1 fragment of the strain 160/2015/ITA (GenBank accession no. MT00824650) [11] into the pCR 2.1 vector of the TA Cloning Kit (Invitrogen, Ltd., Milan, Italy). To prevent any contamination occurring during the quantitative RT-PCR assay, three negative controls containing all of the necessary components of the RT-PCR mixtures, except for the template RNA, were included in each run.

### 2.3. Immunohistochemistry (IHC) Assay

Sections of 5 µm were cut from the formalin-fixed and paraffin-embedded (FFPE) skin biopsies of the footpad ulcerative lesion and mounted on positive-charged slides. Immunohistochemistry was performed using a rabbit polyclonal antibody raised against the FCV-F9 vaccine strain [37]. Briefly, the FFPE tissue sections were deparaffinized, rehydrated, and washed in distilled water. Endogenous peroxidase was blocked with 3% H_2_O_2_ for 8 min at room temperature. The slides were then washed in Tris Buffered Saline (TBS) (Invitrogen Ltd., Milan, Italy) and incubated with the primary antibody for 1 h in a humidified chamber at room temperature. The slides were incubated with secondary biotinylated universal secondary antibody, which was followed by sequential incubation with peroxidase-labelled streptavidin (LSAB+/System-HRP, Dako, Glostrup, Denmark). The negative control was treated in the same manner, omitting the primary antibody, and incubating the tissue sections with TBS. 

### 2.4. Whole Genome Sequencing (WGS) and Phylogenetic Analysis 

The oropharyngeal swab, and the foot skin and blood samples from cat #9284-5, and the oropharyngeal specimen collected from the cohabiting cat (#9341-1) were subjected to a sequence-independent single-primer amplification (SISPA) enrichment, as previously described [38,39,40], and to deep sequencing to generate the complete genome of the FCV strain. The PCR-enriched samples were cleaned up with AMPure XP beads (Beckman Coulter, Brea, CA, USA), quantified by a Qubit dsDNA HS assay (Thermo Fisher Scientific, Waltham, MA, USA), and used for the libraries preparation and for adapter ligation by the Ligation kit SQK-LSK110 (Oxford Nanopore Technologies, ONT, Oxford, UK) following manufacturer’s guidelines. Each test was run for nine hours on an Oxford Nanopore MinION Mk1C device using FLO-MIN106 R9.4.1 flow cell after loading 75 μL of the sequencing mix (12 μL library, 25.5 μL loading beads II, and 37.5 μL sequencing buffer II). The ONT MinKNOW software (v3.1.5) (Oxford Nanopore Technologies, ONT, Oxford, UK) was used to collect the raw sequencing data, and ONT cloud-based basecaller based on GUPPY (v3.2.8) (Oxford Nanopore Technologies, ONT, Oxford, UK) was used to perform the on-site and real-time basecalling during the sequencing run. Sequence trimming, the assembly of reads, and genome annotation were performed using the Geneious Prime version 2021.1.1 (Biomatters Ltd., Auckland, New Zealand). The FCV-F9 strain (GenBank accession no. M86379) was used to create a reference-based assembly by Minimap2. Open reading frame (ORF) prediction and annotations were performed in Geneious Prime software v. 2021.2.2 (Biomatters Ltd., Auckland, New Zealand), and the alignment of the sequences was conducted using Multiple Alignment based on Fast Fourier Transform (MAFFT) [41]. The phylogenetic analysis was performed in MEGA X software [42].

### 2.5. Virus Isolation and Phenotypic Characterization 

The Crandell-Reese feline kidney (CRFK) [43] cell line was cultured in Dulbecco’s modified Eagle’s medium (DMEM) supplemented with 10% heat-inactivated foetal bovine serum. Attempts to conduct the virus isolation were performed as previously described [44]. Each isolate was purified for three times by a plaque assay, which was followed by the preparation of virus stocks obtained by the inoculation of the CRFK cells at a multiplicity of infection of 0.1. The suspensions were harvested after 24 h by three cycles of freezing and thawing and clarified by centrifugation at 1800 rpm for 10 min. The resulting titre was calculated by the Reed and Muench endpoint method, which identified the final infectivity at 50% tissue culture infectious doses (TCID50). All of the isolates were assessed to investigate the in vitro stability when they are exposed to a low pH (3.0), trypsin (final concentration of 0.5%), and to a bile salts (final concentration of 0.5%) treatment using protocols that have been previously described [11]. 

### 2.6. Seroneutralization Assays

The serum samples collected from both of the cats (#9284-5 and #9341-1) were tested for the presence of neutralizing antibodies against the FCV-F9 vaccine strain by performing seroneutralization (SN) assays as previously described [45]. Briefly, 50 μL of each serial twofold diluted serum and 50 μL of infectious culture medium containing 100 TCID50 of the FCV-F9 strain were mixed and incubated for 1 h at 37 °C. One hundred microliters (1 × 10^5^ cell/mL) of CRFK cell suspension were added to each well. The assay was performed in triplicate for each serum. After 3 days of incubation at 37 °C with 5% CO_2_, the end point titers were determined as the highest serum dilution that was able to neutralize the cytopathic effect of the virus. To investigate the antigenic correlation between the field isolates and the vaccine strain, the rabbit polyclonal antibody raised against the FCV-F9 [37] was tested for its neutralizing activity against the FCV strains isolated either from the affected cat or from the asymptomatic co-habiting animal following the procedure described above. An internal FCV-F9 homologous control was included in each experiment.

## 3. Results

### 3.1. Molecular Identification

By using a nested RT-PCR protocol [32] that was able to amplify a short diagnostic genome fragment (477-nt) of the ORF2 region of FCV, viral RNA was detected in the skin, blood, oropharyngeal, and enteric samples collected from the cat #9284-05, whilst all of the specimens tested negative for FHV-1, FPV, FCoV, FeLV, and FIV, thereby ruling out mixed infections. The subsequent RT-qPCR [35,36] revealed viral loads ranging from 1.1 × 10^1^ to 4.1 × 10^5^ RNA copies/5 μL of the RNA template (Table 1), with the highest titers being found, respectively, in the oropharyngeal swab (4.1 × 10^5^) and in the skin sample (2.6 × 10^5^). The FCV RNA at the viral load of 3.8 × 10^4^ RNA copies/5 μL was also detected in the oropharyngeal specimen collected from the healthy cohabiting cat (#9341-1). All of the negative controls did not produce any detectable fluorescence signal.

### 3.2. IHC Identification

**Figure 2:**
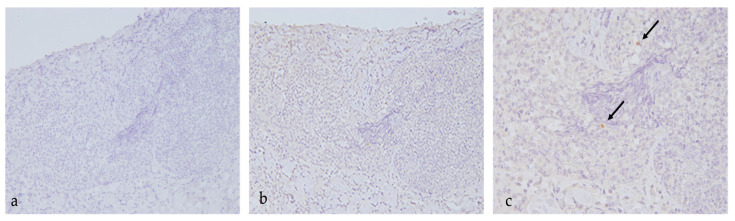
IHC on skin biopsy of the footpad ulcerative lesion using a rabbit polyclonal antibody raised against the FCV-F9 vaccine strain [37]. (**a**) (20X): negative control; (**b**) (20X), (**c**) (40X): positive cells. Arrows indicate positive histiocytes.

By performing IHC staining on the footpad skin lesion sample, scattered positive cells were evident in the deep lympho-histiocyticinflammatory infiltrate, confirming the presence of the FCV antigen (Figure 2).

### 3.3. Molecular Characterization

The combination of an SISPA approach with an ONT sequencing platform allowed us to reconstruct the full-length genome sequences, encompassing the entire ORF1, ORF2, and ORF3 genes of strains 9284-5/2020/ITA detected in the oropharyngeal swab (OS) and skin (S) samples. Additionally, the ONT platform was successfully used to generate the complete genome of the strain 9341-1/2020/ITA identified in the oropharyngeal swab from the asymptomatic cohabiting cat. The complete genomes of FCV-S 9284-5/2020/ITA generated from the skin and of FCV-OS 9341-1/2020/ITA identified in the oropharyngeal swab were deposited in GenBank under the accession numbers OP750454 and OP750455, respectively. On pairwise homology and distance analyses, strains FCV-OS 9284-5/2020/ITA and FCV-OS 9341-1/2020/ITA displayed a nucleotide (nt) identity of 99.9% to each other with a deduced amino acid (aa) identity of 100% either in the polyprotein and in the structural proteins VP1 and VP2.A single aa substitution in position 432 (Ala-432-Val) was observed in the capsid protein of the skin virus isolate FCV-S 9284-5/2020/ITA.

**Figure 3:**
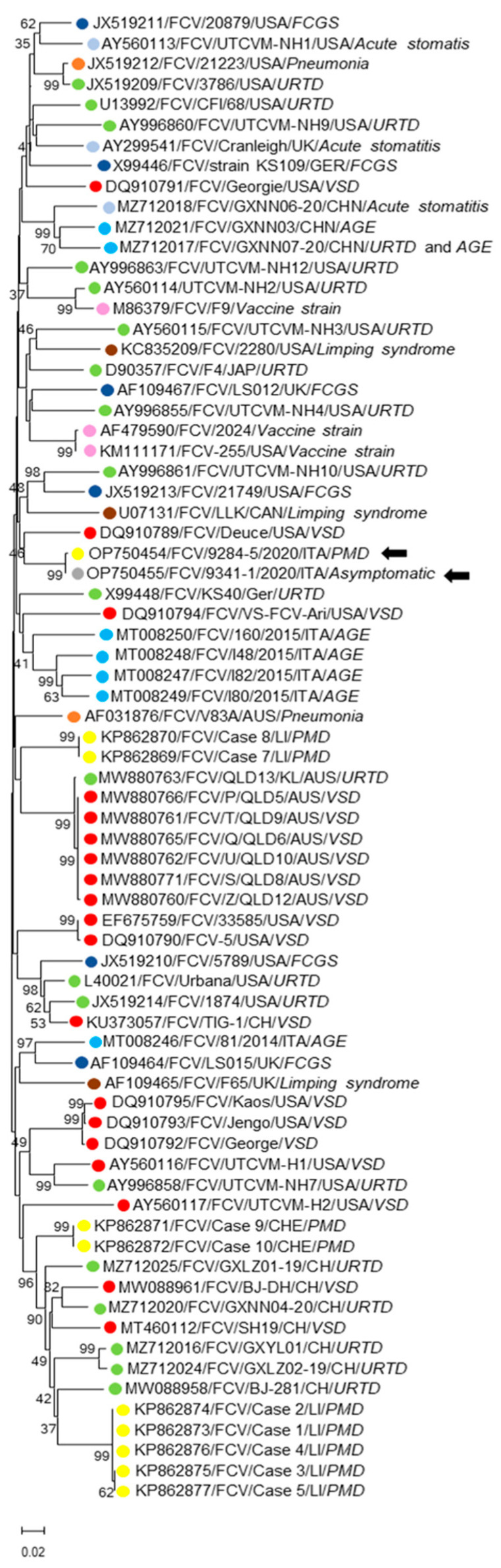
Phylogenetic analysis based on the aa sequence of the full-length VP1 capsid protein was generated using the maximum likelihood. The evolutionary distances were computed using the Poisson correction method and are in the units of the number of aa substitutions per site, supplying statistical support with bootstrapping of 1000 replicates. Arrows indicate the FCV strains detected in this study. Abbreviations: VSD = virulent systemic disease; PMD = paw and mouth disease; URTD = upper respiratory tract disease; FCGS = feline chronic gingivostomatitis; AGE = acute gastroenteritis. Disease phenotype is colour coded.

Upon the preliminary analyses with BLAST (www.ncbi.nlm.nih.gov/blast, accessed on 10 September 2022) and FASTA (www.ebi.ac.uk/fasta33, accessed on 10 September 2022), the overall nt identity similarities of the FCV-OS strains 9284-5/2020/ITA and 9341-1/2020/ITA to the FCV complete sequences currently available in the databases ranged from 76.2% to 81.2%. The distance and maximum likelihood-based phylogenetic analyses were performed using a selection of VP1 capsid sequences consisting of three FCV vaccine strains (FCV F9, FCV 255, and FCV 2024) and 68 field strains retrieved from GenBank and isolated from distinct disease manifestations. In the VP1 aa sequence, the similarities of the identities of strains 9284-5/2020/ITA and 9341-1/2020/ITA to other FCVs were 83.1–91.3%, with the highest identity being attributed (91.1–91.3%) to a VSD isolate (FCV/Deuce/USA/VSD DQ910789) [18]. The overall similarities of the aa identities of all of the FCV capsid sequences available in the databases ranged from 81.6% to 92.3%. The similarities of the identities of the two Italian strains to FCV sequences associated with PMD, VSD, and upper respiratory tract disease (URTD) greatly overlapped with each other, with values, respectively, of 85.2–90.4%, 86.3–91.3%, and 85.5–90.5%. Additionally, upon visual inspection of the alignments, the aa changes unique to the PMD or VSD isolates were not found. In the VP1-based tree (Figure 3), the FCVs were grouped into several polyphyletic clades, without any evidence of clustering patterns correlated to a clinical disease. The strains 9284-5/2020/ITA and 9341-1/2020/ITA were grouped alone with FCV/Deuce/USA (DQ910789), although not tightly. A similar phylogenetic topography was also observed in the tree performed only on the region E (aa 426-523) (data not shown) of the VP1 protein.

To further investigate the possible genetic relationship of our strains with VSD-FCVs, the inferred aa sequences of the hypervariable region E of the Italian viruses were mapped to identify seven aa residue positions (438, 440, 448, 452, and 455, 465, and 492), whose physical and chemical properties were previously shown to be statistically significant for differentiation between the URTD and VSD FCV pathotypes [46]. The alignments were performed, including the corresponding sequences extracted from nine FCV strains previously detected in cats showing paw and mouth signs (KP862869–KP862877) [27] (Table 2). In our analysis, the predicted properties for the virulent pathotypes were found in five of the seven residues (positions 438, 440, 452, 455, and 492) of the hypervariable region E of both of the strains 9284-5/2020/ITA and 9341-1/2020/ITA and in only three positions of the paw and mouth disease FCVs.

### 3.4. Biochemical and Antigenic Characterization

FCV was successful isolated from the skin and oropharyngeal samples of the affected cat (#9284-5), as well as from the oropharyngeal swab of the asymptomatic cat (#9341-1). The three isolates were titrated and analyzed to investigate their in vitro sensitivity to the pH, bile salts, and trypsin treatments. As reported in Table 3, all of the isolates were completely resistant at pH 3.0, and they were slightly susceptible to the bile salts, with a titres reduction of 1.25 log10, whilst after exposure to trypsin, a decrease in the titres of 3.0 log10 was observed. By assessing the serum samples from both of the cats by the SN assay, specific neutralizing antibodies anti-FCV-F9 were detected, respectively, at titers of 1:64 in the affected cat and 1:128 in the cohabiting healthy animal. When we tested the anti-FCV-F9 serum against the three isolates, all of the strains were neutralized at titres of 1:8, while the neutralizing activity against the homologous virus occurred at 1:256.

## 4. Discussion

The high degree of genomic plasticity of FCV accounts for the emergence of several variants, some of which are associated with a severe clinical disease. This is exemplified by the increasing number of reports on VSD-FCVs, which are divergent from the “classical mild FCVs” for their marked tissue tropism for epithelial and endothelial cells, multisystemic target, the induction of systemic vascular failure, and for involvement of visceral organs [12,13,14,15,16,17,18,19,20,21,22,23]. In contrast, FCV-associated PMD has been described on only a few occasions [24,25,26,27], and it is still unclear whether these cases represented mild forms of VSD or they were rather the clinical presentation of a distinct syndrome. 

In this study, we documented a case of FCV infection in a 7-year-old cat showing ulcerative lesions on the skin of their paws and in and around the mouth, but this occurred without inner organs involvement. The presence of the FCV antigen in the skin lesion was confirmed by the IHC assay. Furthermore, the molecular screening by qualitative and quantitative RT-PCRs detected FCV RNA in all of the tested specimens, with the highest viral RNA loads being in the oropharyngeal and skin samples, and the lowest ones being in blood and rectal specimens. A high viral load was also found in the oropharyngeal specimen collected from a clinically healthy cat living in contact with the animal with PMD. After the complete genome analysis, the viruses of oropharyngeal origin identified in the two cats were identical to the deduced aa sequence (100% aa identities in polyprotein, VP1 and VP2), whilst the isolate detected in the skin sample differed for a single aa substitution (432) which was located in the 5′ hypervariable region E (426–460 aa residues) of the VP1. Attempts were made to investigate the genetic relationship between the FCV strains detected in this study and FCV strains previously found in association with PMD, VSD, URTD, acute and chronic gingivostomatitis, limping syndrome, and acute gastroenteritis. As expected, the VP1-based phylogenetic analysis failed to identify the well-defined pathotype-associated clusters. The intrinsic molecular mechanisms by which some FCV isolates show a highly virulent phenotype are still unclear. The analysis of the FCV sequences from VP1 region E has been used to differentiate VSD from classical respiratory FCVs, and we have identified seven key residue positions (438, 440, 448, 453, 455, 465, and 492) which are statistically significant for pathotype differentiation, and these are mainly located in the N-terminal hypervariable part of region E [46]. This domain displays the highest variability along the VP1 protein, and it includes immunodominant neutralizing epitopes that are able to interact with the feline cell receptor JAM-1 during the viral attachment [47,48,49,50,51,52]. In our analysis, after the residue mapping of the hypervariable region E of the strains 9284-5/2020/ITA and 9341-1/2020/ITA, only five of the seven key residue positions showed the predicted physical and chemical properties. Likewise, none of FCV strains previously detected in the cats with suspected PMD [27] showed the VSD combination pattern. 

Both of the strains (9284-5/2020/ITA and 9341-1/2020/ITA) detected in the oropharyngeal swabs and in the footpad skin sample (9284-5/2020/ITA) were adapted to grow in the CRFK line, while the viruses identified from the blood and rectal swabs could not be adapted to replicate in vitro. This could be accounted for by the low viral loads found in the faecal swab (1.2 × 10^2^) and the blood sample (1.1 × 10^1^). There is evidence that FCVs associated with atypical clinical signs possess in vitro phenotype properties that are different from FCV strains which are responsible for the classical respiratory disease [11,18,53]. The in vitro characteristics of the three isolates obtained in this study revealed a high resistance to a low pH treatment and only a moderate susceptibility to the bile salts, yet, they were susceptible to the trypsin treatment, differing in their phenotype patterns from FCV strains of respiratory origin. Cats #9284-5 and #9341-1 had been immunized against FCV with a vaccine based on the live attenuated strain F9, although the vaccination dated back to 2018, i.e., 3 years before the PMD case. Neutralizing antibodies versus FCV F9 were detected in the serum collected from both of the animals at titers, respectively, of 1:64 in the PMD cat and of 1:128 in the cohabiting healthy animal. However, when we were testing the neutralizing activity of an F9 antisera, all of the three isolates were neutralized at titers of 1:8, suggesting a low antigenic cross-reactivity between the field strains obtained in this study and the vaccine FCV-F9. Additionally, these findings confirmed the remarkable variability of FCVs in terms of the virus neutralization profile [27,54].

In the present case report, the origin of the infection remains unknown since both of the cats were exclusively kept indoors. However, the contact cat was hospitalized for an orthopedic problem two weeks before the onset of the clinical signs in cat #9284-5. Accordingly, we speculated that cat #9341-1 could have acquired an asymptomatic infection during hospitalization, acting as carrier for FCV.

## 5. Conclusions

We described a rare case of PMD due to an FCV infection in a vaccinated cat that received its last booster 3 years before. The same virus was detected in a contact animal that was overtly healthy and that likely introduced the virus in the household after a nosocomial inapparent infection. Genetic hallmarks accounting for the PMD clinical form were not found in PMD-associated FCVs, although the FCV strain sequenced from the skin lesions differed in a unique aa mutation (Ala-432-Val) in the hypervariable region of the capsid. The whole genome sequencing of virus pairs or clones, i.e., viral strains obtained from different lesions/tissues of the same animal, could help us to decipher the mechanisms driving phenotype shifts in FCV.

## Figures and Tables

**Table 1:** Quantification of FCV RNA by qRT-PCR in samples collected from cats #9284-5 and #9341-1.

Cat ID	Sample	qRT-PCR (Ct)	RNA Copies/5 μL
#9284-5	Oropharyngeal swab	13.7	4.1 × 10^5^
	Footpad skin sample	15.4	2.6 × 10^5^
	Rectal swab	30.4	1.2 × 10^2^
	Blood	33.6	1.1 × 10^1^
#9341-1	Oropharyngeal swab	20.7	3.8 × 10^4^

**Table 2:** Residues of region E of the VP1 capsid protein statistically associated with the VSD-FCV pathotype [46].

	Physicochemical Properties of Amino Acids Associated with VSD Pathotype						
Strain	438	440	448	452	455	465	492
	Non Polar, Aliphatic Chain	Non Small	Polar, Positive Charged	Non Small	Non Negative	Polar	Small
VSD FCV	V_9_T_8_	Q_6_G_5_E_4_SK	K_7_A_2_E_2_G_2_P_2_R_2_	E_11_D_6_	T_6_D_4_M_2_I_2_NES	S_14_G_3_	V_16_R
URTD FCV	T_37_V_2_I	G_22_S_6_Q_4_R_2_A_2_ENDT	A_30_P_4_G_3_K_3_	D_36_E_3_N	D_27_T_5_S_3_V_2_GRE	G_26_S_14_	V_18_L_8_I_7_R_5_K_2_
9284-5/2020/ITA/*PMD* (OP750454)	**V**	**H**	L	**E**	**I**	G	**V**
9341-1/2020/ITA/ *Asyntomatic* (OP750455)	**V**	**H**	L	**E**	**I**	G	**V**
Case 1/LI/*PMD* (KP862873)	T	**E**	P	D	**K**	**S**	I
Case 2/LI/*PMD* (KP862874)	T	**E**	P	D	**K**	**S**	I
Case 3/LI/*PMD* (KP862875)	T	**E**	P	D	**K**	**S**	I
Case 4/LI/*PMD* (KP862876)	T	**E**	P	D	**K**	**S**	I
Case 5/LI/*PMD* (KP862877)	T	**E**	P	D	**K**	**S**	I
Case 7/LI/*PMD* (KP862869)	**I**	G	**R**	**E**	D	G	L
Case 8/LI/*PMD* (KP862870)	**I**	G	**R**	**E**	D	G	L
Case 9/LI/*PMD* (KP862871)	T	G	P	D	**A**	**S**	**V**
Case 10/LI/*PMD* (KP862872)	T	G	P	D	**A**	**S**	**V**

Residues in bold indicate amino acids matching with the VSD configuration.

**Table 3:** Titre of FCV (log10 TCID50) and log10 reduction obtained after application of different treatments.

	Oropharyngeal Swab 9284-5/2020/ITA		Footpad Skin Sample 9284-5/2020/ITA		Oropharyngeal Swab 9341-1/2020/ITA	
	Control	Treated	Control	Treated	Control	Treated
HCl—pH3	7.25	7.25	7.25	7.25	7.25	7.25
Bile salts (0.5%)	7.25	6.0	7.25	6.0	7.25	6.0
Trypsin (0.5%)	7.25	4.25	7.25	4.25	7.25	4.25

## Data Availability

The data presented in this study are available in this manuscript. Sequence data presented in this study are openly available in the GenBank database.
